# Anti-cancer efficacy including *Rb*-deficient tumors and *VHL*-independent HIF1α proteasomal destabilization by dual targeting of CDK1 or CDK4/6 and HSP90

**DOI:** 10.1038/s41598-021-00150-8

**Published:** 2021-10-22

**Authors:** Shuai Zhao, Lanlan Zhou, David T. Dicker, Avital Lev, Shengliang Zhang, Eric Ross, Wafik S. El-Deiry

**Affiliations:** 1 Laboratory of Translational Oncology and Experimental Cancer Therapeutics, Warren Alpert Medical School, Brown University, Providence, RI USA; 2 Pathobiology Graduate Program, Brown University, Providence, RI USA; 3 Department of Pathology and Laboratory Medicine, Brown University, Providence, RI USA; 4 Joint Program in Cancer Biology, Brown University and Lifespan Cancer Institute, Providence, RI USA; 5 Cancer Center at Brown University, Warren Alpert Medical School, Brown University, Providence, RI USA; 6 Fox Chase Cancer Center, Philadelphia, PA USA; 7 Hematology/Oncology Division, Lifespan Cancer Institute, Providence, RI USA

**Keywords:** Cancer, Cell biology, Oncology

## Abstract

A prevalent characteristic of solid tumors is intra-tumoral hypoxia. Hypoxia-inducible factor 1α (HIF1α) predominantly mediates the adaptive response to O_2_ oscillation and is linked to multiple malignant hallmarks. Here we describe a strategy to robustly target HIF1α by dual inhibition of CDK(s) and heat shock protein 90 (HSP90). We show that CDK1 may contribute to HSP90-mediated HIF1α stabilization. CDK1 knockdown enhances the decrease of HIF1α by HSP90 inhibition. Dual inhibition of CDK1 and HSP90 significantly increases apoptosis and synergistically inhibits cancer cell viability. Similarly, targeting CDK4/6 using FDA-approved inhibitors in combination with HSP90 inhibition shows a class effect on HIF1α inhibition and cancer cell viability suppression not only in colorectal but also in various other cancer types, including *Rb*-deficient cancer cells. Dual inhibition of CDK4/6 and HSP90 suppresses tumor growth in vivo. In summary, combined targeting of CDK(s) (CDK1 or CDK4/6) and HSP90 remarkably inhibits the expression level of HIF1α and shows promising anti-cancer efficacy with therapeutic potential.

## Introduction

Accompanying the unrestrained proliferation of malignant cells, solid tumors are generally deprived of an adequate oxygen supply^1^. Regions located further than the oxygen diffusion limit (~ 100 μm)^2^ to blood vessels become hypoxic. Hypoxia is implicated in cancer, linked to abnormal vascularization, altered metabolism, resistance to chemo-/radio-therapy, as well as increased cancer cell stemness and metastasis^3–7^. In adaptation to hypoxia, hypoxia-inducible factor 1 (HIF1), as a transcription factor, stimulates a variety of target genes that are involved in altered metabolism, cell survival and tumor progression^8–10^. In particular, the α subunit of HIF1, HIF1α, becomes constitutively expressed, which leads to the constant activation of HIF1.

Overexpression of HIF1α is observed in a variety of cancers. In colorectal cancer (CRC), it is associated with poor prognosis and early progression^11,12^. HIF1α inhibits apoptosis^13,14^, facilitates cell migration^14^ and promotes angiogenesis through upregulation of the target *VEGF* gene^15^ in CRCs and other tumors. When oxygen is sufficient, in normal cells, HIF1α is hydroxylated by prolyl hydroxylase-domain proteins (PHDs) and is targeted by the von Hippel-Lindau (VHL) protein complex for ubiquitination and subsequent proteasomal degradation^16^, which is prevented by hypoxia^17^. However, elevated HIF1α expression is not exclusive to hypoxic conditions. In renal cell carcinomas, VHL is frequently mutated and deficient^18^. In RCC4 renal cancer cells, for instance, HIF1α is constantly expressed at increased levels due to protein stabilization. EGF/EGFR signaling transcriptionally activates HIF1α independently of hypoxia^19^. Moreover, HIF1α was shown to be detectable at other regions in the tumor other than the hypoxic necrotic margin^20^. HIF1α can accumulate in T_H_17 cells under normoxia and regulates T_H_17 differentiation, suggesting a role of HIF1α in the immune system in both normoxia and hypoxia^21^.

We previously carried out a chemical library screen for hypoxia sensitizers and uncovered cyclin-dependent kinase inhibition as a potential target^22^. We further showed that cyclin-dependent kinase 1 (CDK1) stabilizes HIF1α through phosphorylation of the Ser668 residue of HIF1α protein^23^. Such stabilization occurs not only in hypoxia, but also at the G2/M cell cycle phase under normoxic conditions. Moreover, CDK4 is also important for HIF1α stabilization, as we uncovered in our study through knockdown of CDK proteins^23^. Assessment of RCC4 cells demonstrated that the CDK1- or CDK4/6- inhibitor-mediated HIF1α destabilization is independent of a functional VHL protein.

Another VHL-independent HIF1α stabilizer is the heat shock protein 90 (HSP90)^24^. HSP90 is a HIF1α-associated protein^25^. Overexpression of HSP90 has been correlated with adverse prognosis and recognized as a therapeutic target in cancer (e.g. esophageal squamous cell carcinoma, melanoma, leukemia)^26–28^. Both CDK and HSP90 inhibitors have been widely studied^29,30^. Ro-3306 is a CDK1-selective inhibitor^31^. The CDK4/6 inhibitor, palbociclib, among others has been approved by the FDA in combination treatment for breast cancers^32,33^. HSP90 inhibitors have evolved from the classic geldanamycin to second generation compounds (e.g. ganetespib^34^). In colorectal cancer, ganetespib has been found to inhibit angiogenesis^35^ and sensitizes cells to radiation and chemotherapy^36^.

We investigated the hypothesis that CDK1 and HSP90 signaling overlaps in the regulation of HIF1α, and combination treatment to target both CDK1 and HSP90 may lead to enhanced inhibitory effects towards HIF1α expression and function as well as improved anti-cancer efficacy. We extended our observations to CDK4/6 inhibitors given the fact there are several FDA-approved drugs allowing more rapid translation of our findings. We uncovered a synergy between CDK4/6 inhibitors and HSP90 inhibitors, as a class effect for each of the two drug classes, through convergence upon HIF1α leading to cell death. Importantly, the dual blockade of CDK4/6 and HSP90 is observed in *Rb*-deficient tumor cells suggesting a novel approach for cancer therapy. An additional aspect of this work involves a focus on HIF as a target and potential biomarker for CDK4/6-HSP90 dual inhibition therapy, that could be further tested in clinical trials.

## Results

### CDK1 contributes to HSP90-mediated HIF1α stabilization

**Figure 1:**
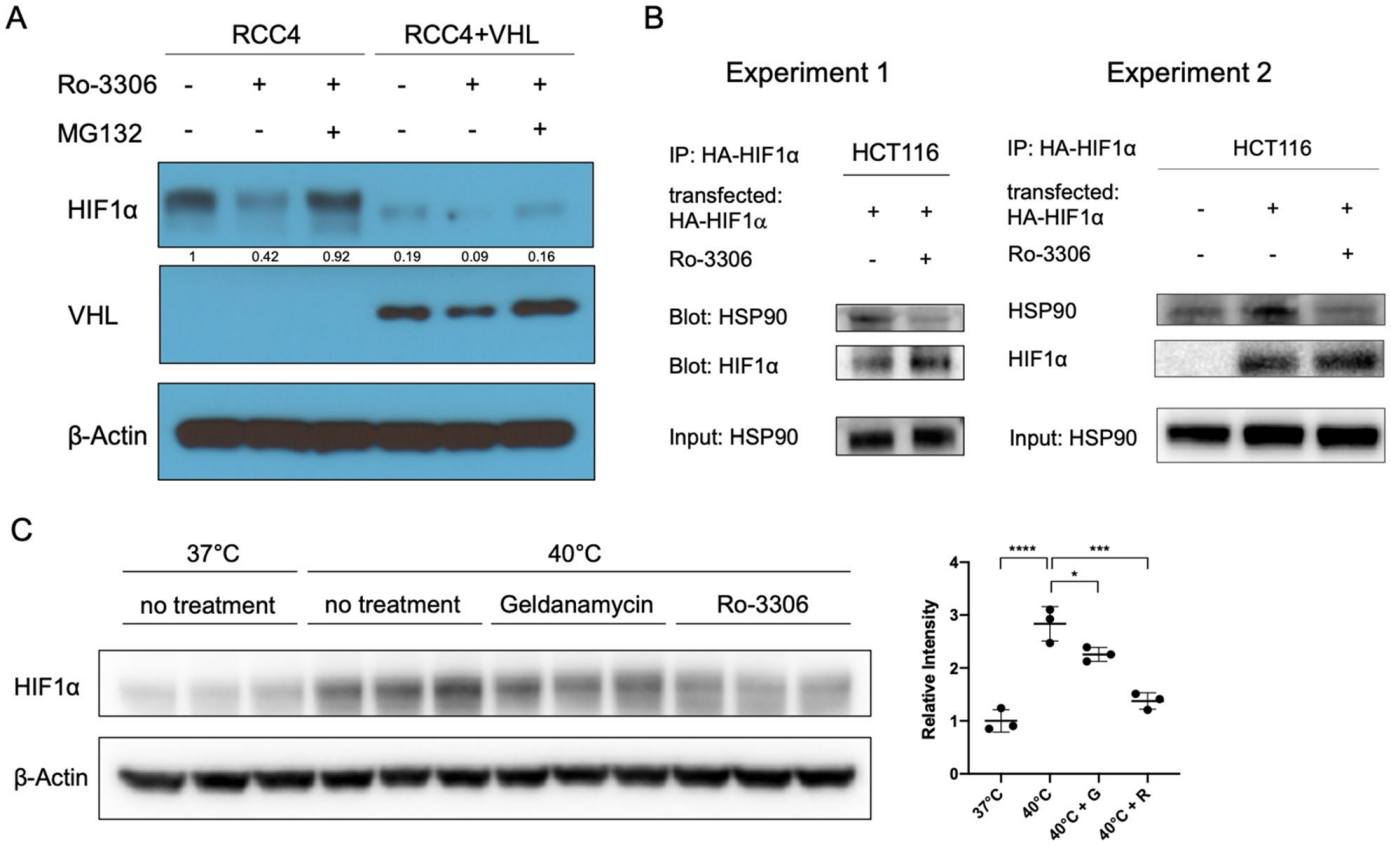
CDK1 contributes to HSP90-mediated HIF1α stabilization. (**A**) Inhibition of CDK1 decreases the level of HIF1α in RCC4 cells independently of *VHL*. Cells were treated with Ro-3306 (5 μM) or MG132 (1 μM) or both as indicated for 6 h under normoxia. (**B**) CDK1 inhibition (for 6 h under hypoxia; 0.5% O_2_) impairs the interaction between HIF1α and HSP90. HCT116 cells were treated with MG132 and cultured in hypoxia for 6 h with or without Ro-3306. Cells were fixed and lysed for co-immunoprecipitation analysis. Experiment 2 is a repeated experiment performed under the same conditions as in Experiment 1, with an added control (without HA-HIF1α overexpression). (**C**) CDK1 inhibition partially reversed heat shock-induced HIF1α expression. HCT116 cells were treated at 40 °C with the indicated inhibitors for 6 h. Geldanamycin was used at 2 μM. Intensity was measured compared to β-Actin in each lane. G: geldanamycin; R: Ro-3306. Statistical analysis was performed using one-way ANOVA supplemented with Tukey test. Mean ± SD was shown. *p < 0.05, **p < 0.01, ***p < 0.001, ****p < 0.0001.

We have previously reported that knockdown of CDK1 led to the reduction of HIF1α level in RCC4 VHL-deficient cells^23^. To reinforce the hypothesis that the regulatory effect on HIF1α by CDK1 inhibition is independent of VHL, we examined the level of HIF1α upon addition of CDK1 inhibitor, Ro-3306, in both RCC4 and RCC4^VHL+^ cells. As expected, HIF1α was constantly expressed in RCC4 cells under normoxia, owing to the loss-of-function mutation of *VHL* in this cell line. In accordance with previous results, CDK1 inhibition reduced HIF1α level in RCC4 in normoxia, which could be reversed by proteasome inhibition with MG132 (Fig. 1A). In RCC4^VHL+^ cells where *VHL* is stably reintroduced, HIF1α expression was dramatically decreased in normoxia compared to that in RCC4 cells. Inhibition of CDK1 decreased the level of HIF1α in RCC4^VHL+^ cells, which could be rescued with MG132 (Fig. 1A). Thus, CDK1 inhibition destabilized HIF1α in a *VHL*-independent manner.

Another previously known *VHL*-independent HIF1α stabilizer and associating partner is HSP90^24,25^. We asked whether there is a link between CDK1-mediated and HSP90-mediated stabilization of HIF1α. We found that inhibition of CDK1 impaired the interaction between HIF1α and HSP90 (Fig. 1B). Moreover, heat shock (40 °C) induced HIF1α expression in normoxia, which could be partially reversed by treatment with HSP90 inhibitor, geldanamycin, or CDK1 inhibitor, Ro-3306 (Fig. 1C). These results suggest that CDK1 may contribute to the stabilization of HIF1α by HSP90.

### Dual targeting of CDK1 and HSP90 robustly reduces the expression level of HIF1α

**Figure 2:**
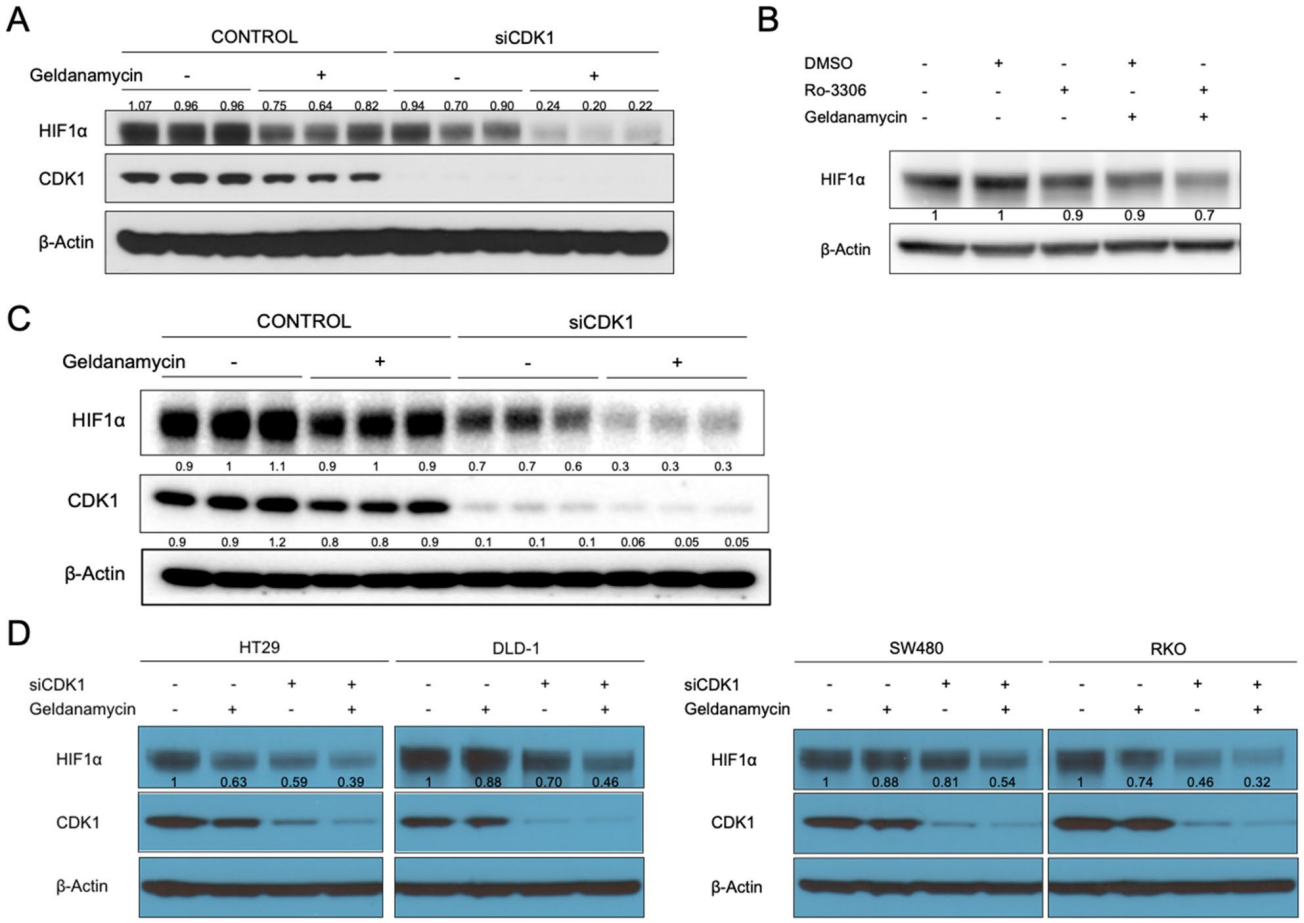
Dual inhibition of CDK1 and HSP90 robustly reduces the level of HIF1α. (**Α**) HCT116, (**C**) HCT116 *p53*^−/−^ cells or (**D**) other colorectal cell lines were treated with control or CDK1 siRNA for 48 h, followed by treatment with DMSO or geldanamycin (2 μM) under hypoxia (0.5% O_2_) for 6 h. (**B**) Cells were treated with Ro-3306, geldanamycin, or the combination of both for 6 h under hypoxia.

On basis of the findings above, we tested whether targeting CDK1 could enhance the inhibitory effect on HIF1α expression by HSP90 inhibitors. Consistent with previous findings, the level of hypoxia-induced HIF1α was decreased by CDK1 knockdown or HSP90 inhibition with geldanamycin. Remarkably, when geldanamycin was added to CDK1-knockdown cells, the reduction of HIF1α was further enhanced (Fig. 2A). Such enhanced HIF1α inhibition was also observed with combination treatment using the two inhibitors, Ro-3306 and geldanamycin (Fig. 2B).

It is known that *p53* is mutated in approximately 40%-50% of sporadic colorectal cancers^37^, we tested whether the absence of p53 affects the combinational effect. In HCT116 *p53*^−/−^ cells, combination treatment robustly diminished the level of HIF1α similarly as in wild-type cells (Fig. 2C), indicating that the HIF1α-regulatory effect is *p53*-independent. Consistently with these observations, the enhanced inhibition of HIF1α by combination treatment was observed in other colorectal cancer cells with different p53 status (Fig. 2D) (i.e. HT29: *p53*^G273A^; DLD1: *p53*^C241T^, SW480: *p53*^G273A&C309T^; RKO: *p53*^wild-type^).

### Dual inhibition of CDK1 and HSP90 synergistically suppresses cancer cell viability

**Figure 3:**
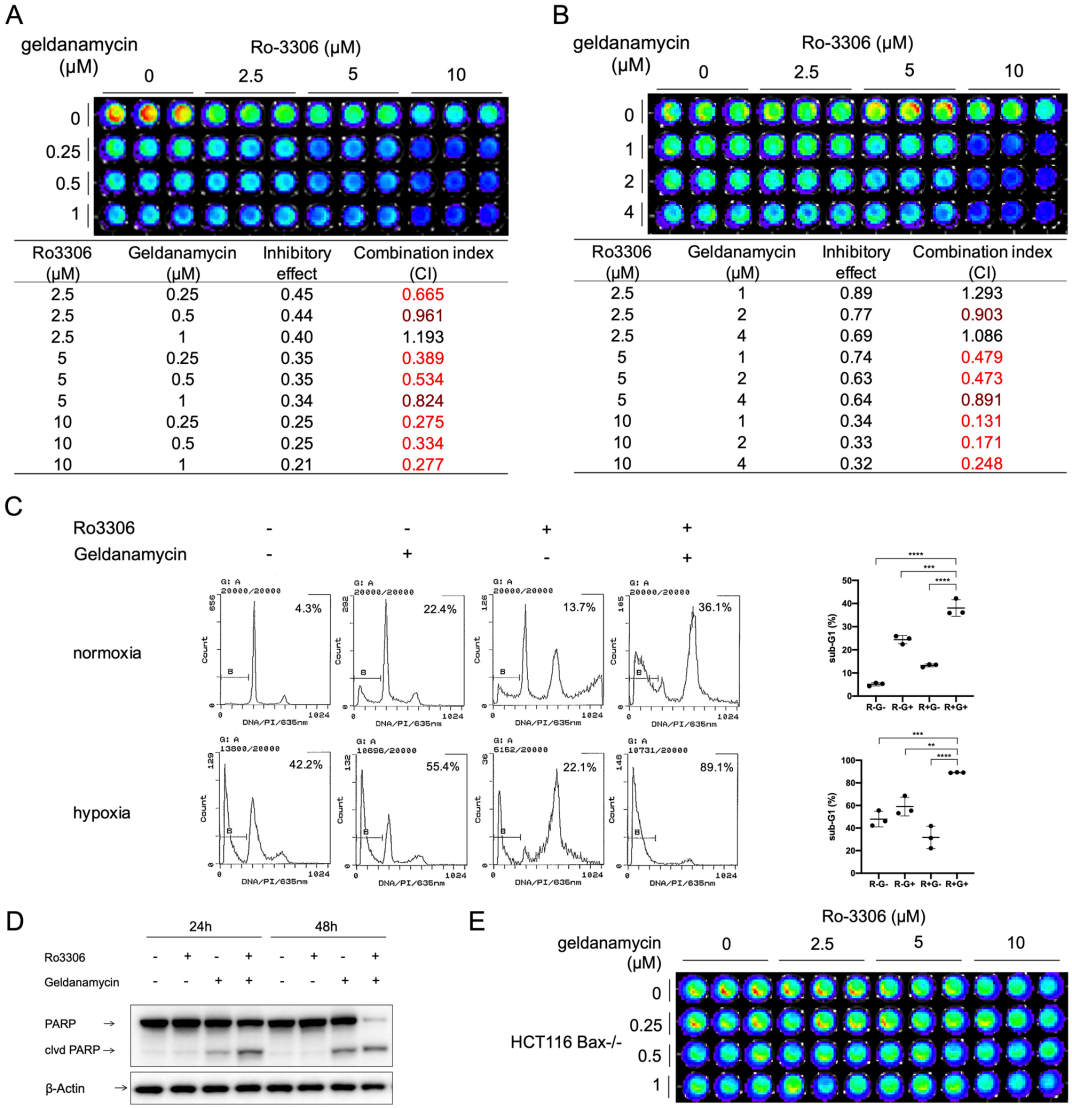
Ro-3306 and geldanamycin synergistically inhibit HCT116 cell viability through induction of apoptosis. (**A**) In normoxia or (**B**) hypoxia (0.5% O_2_), cells were treated with Ro-3306 and geldanamycin at the indicated concentrations for (**A**) 48 or (**B**) 72 h. (**C**) Sub-G1 analysis by propidium iodide staining and flow cytometry of cells treated with Ro-3306 (10 μM) and geldanamycin (1 μM) under normoxia for 48 h or under hypoxia for 72 h. (**D**) Western blot of PARP cleavage in cells treated with Ro-3306 or geldanamycin or both. (**Ε**) CellTiter-Glo analysis of cell viability in HCT116 *Bax*^-/-^ cells treated at indicated concentrations under normoxia for 48 h. Statistical analysis was performed using one-way ANOVA supplemented with Dunnett test. Mean ± SD was shown. *p < 0.05, **p < 0.01, ***p < 0.001, ****p < 0.0001.

The universal effect of HIF1α inhibition by combination of CDK1 knockdown and HSP90 inhibition among various colorectal cancer cell lines prompted us to investigate the therapeutic potential of such combination strategy. We performed a CellTiter-Glo assay to assess the combinatorial effect on cell viability by CDK1 and HSP90 inhibitors. We found that Ro-3306 and geldanamycin synergistically inhibited HCT116 cell viability in both normoxia and hypoxia (Fig. 3A, B).

Subsequently we asked whether apoptosis was induced by the combination treatment. We performed sub-G1 analysis by flow cytometry to estimate fractional DNA content^38^. Combination of Ro-3306 and geldanamycin significantly increased the sub-G1 population in HCT116 cells as compared to control and single treatments in either normoxia or hypoxia (Fig. 3C), indicating the increased occurrence of apoptosis. As expected, PARP cleavage was also observed (Fig. 3D) at an earlier time point as a marker of initiated apoptosis^39^. In addition, the robust synergy on cell viability inhibition was abrogated in HCT116 *Bax*^-/-^ cells (Fig. 3E), indicating that Bax may play an important role in mediating cell death induced by the CDK1i/HSP90i combination treatment.

### Dual inhibition of CDK1 and HSP90 represses the ability of colony formation and cell migration

**Figure 4:**
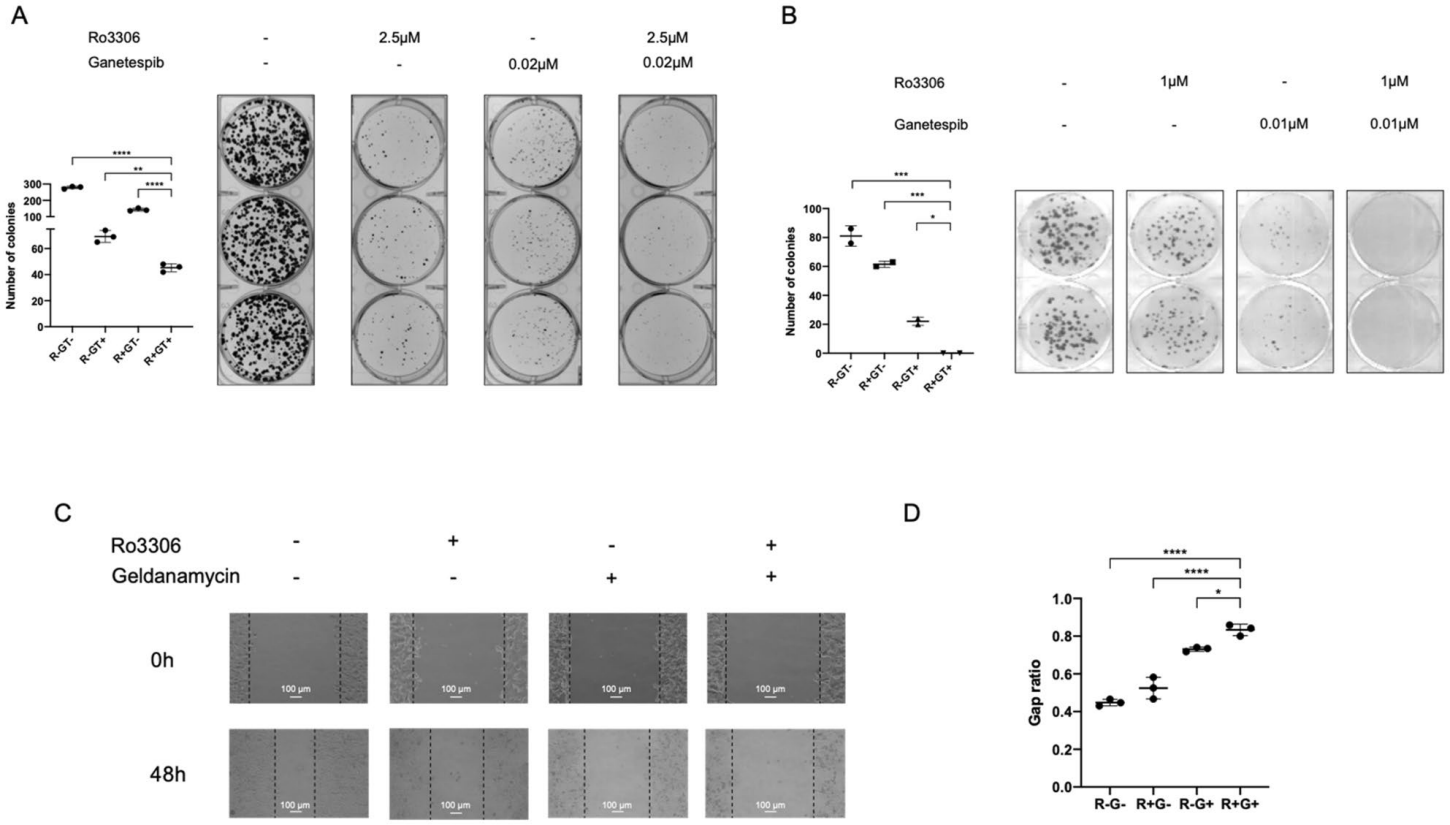
Combination CDK1 and HSP90 inhibitor treatment inhibits colony formation and migration in HCT116 cells. (**A**) In normoxia or (**B**) In hypoxia (0.5% O_2_), HCT116 cells were treated with the indicated combination treatments for 72 h. Drug-containing media was replaced with regular culture media, and cells were allowed to grow and form colonies for 1 week. (**C**) Scratch assay and (**D**) Quantification for HCT116 cells under normoxia for 48 h. Gap ratio refers to the ratio of gap width at 48 h versus at 0 h. Cells were treated with Z-VAD caspase inhibitor to prevent cell death. (R: Ro-3306; G: geldanamycin.) n = 3. Statistical analysis was performed using one-way ANOVA supplemented with Dunnett test. Mean ± SD was shown. *p < 0.05, **p < 0.01, ***p < 0.001, ****p < 0.0001.

Not every single cancer cell is capable of proliferating into a colony^40^. To determine whether the combination treatment as well induces cell reproductive death in an in vitro model, we performed clonogenic assays to test the post-treatment change in cell capability to generate colonies. Treatment with both Ro-3306 and geldanamycin, at relatively low doses (2.5 μM, 0.02 μM, respectively in normoxia; 1 μM, 0.01 μM, respectively in hypoxia), markedly inhibited colony formation of HCT116 cells in both normoxia (Fig. 4A) and hypoxia (Fig. 4B) (also shown at different doses in Supplementary Fig. S1). The colonies that formed upon combination treatment were fewer in number and smaller in size as compared to control and single treatments. Thus, the dual inhibition of CDK1 and HSP90 inhibits colony formation by HCT116 colon cancer cells.

The overexpression of HIF1α in cancer is implicated not only in promoting cell survival but also in cell migration^41^. We performed an in vitro scratch assay^42^ to test the effect of combination treatment on HCT116 motility. An artificial gap was created on a nearly confluent monolayer of cells. The cell monolayer bearing wounds was treated with single or combination of the two inhibitors, together with Z-VAD-FMK, a pan-caspase inhibitor to prevent treatment-induced cell death. Gap ratio was calculated using gap width at 48 h normalized to that at 0 h. The ratio was significantly higher in the combination treatment group as compared to the control and single treatment groups (Fig. 4C, D), suggesting that the combination of Ro-3306 and geldanamycin inhibits HCT116 cell migration.

### Dual inhibition of CDK4/6 and HSP90 shows anti-cancer effects

We have previously shown that knockdown of CDK4 was able to reduce the level of HIF1α^23^. Thus we extended our observations to CDK4/6 inhibitors, given the fact that there are several FDA-approved drugs allowing more rapid translation of our findings. Palbociclib, abemaciclib and ribociclib are the CDK4/6 inhibitors approved in combination treatments for estrogen receptor positive (ER+) breast cancers. A main issue that emerged in clinical application of CDK4/6 inhibitors is the development of resistance in patients. Mechanisms of resistance have appeared to involve alterations in both upstream and downstream of the CDK4/6 pathway. For instance, amplification and activating mutations in the upstream tyrosine receptor kinases (e.g. FGFR1/2, ERBB2) have been related to the resistance to CDK4/6 inhibition^43–47^. The strategy of targeting those receptor kinases in combination with CDK4/6 inhibition is being investigated. Regarding the downstream resistance mechanisms, mutations in *RB1* accounts for approximately 5–10% of the resistance in patients^47–50^. Rb is the downstream mediator in the CDK4/6 pathway, which functions by binding to E2F and prevent its transcriptional activity. Upon phosphorylation of Rb by CDK4/6, this interaction is disrupted which results in the activation of E2F signaling. In the case of *Rb* deficiency, the inhibition of E2F is absent, which induces a phenotype similar to constitutive activated CDK4/6. It has thus been proposed that *RB1* status should be included as a key biomarker when applying CDK4/6 inhibitors in clinic. It is also imperative to investigate approaches that sensitize *Rb*-deficient cells to CDK4/6 inhibition.

**Figure 5:**
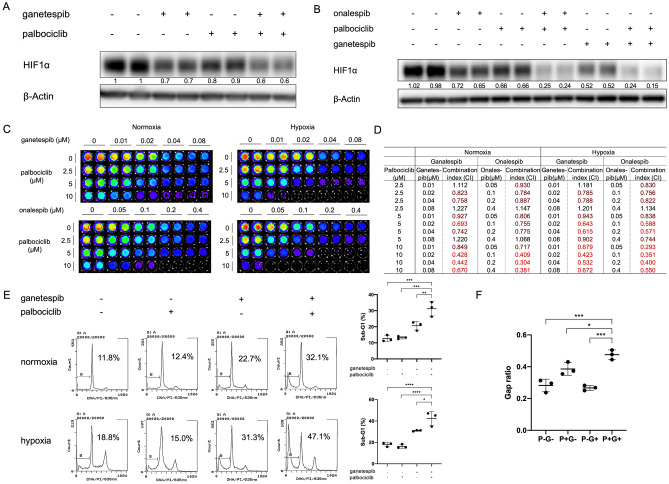
Dual inhibition of CDK4/6 and HSP90 reduces HIF1α in colorectal cancer cells and synergistically inhibits cell viability in HCT116. (**A**) HCT116 cells were treated with the indicated inhibitors (ganetespib at 0.05 μM and palbociclib at 10 μM) for 6 h under hypoxia (0.5% O_2_). (**B**) SW480 cells were treated with the indicated inhibitors (ganetespib at 0.05 μM, onalespib at 0.05 μM, and palbociclib at 10 μM) for 6 h under hypoxia (0.5% O_2_). (**C**, **D**) CDK4/6 inhibitor palbociclib and HSP90 inhibitor ganetespib or onalespib synergistically inhibit the viability of HCT116 cells at 72 h in normoxia and hypoxia (0.5% O_2_). (**E**) Sub-G1 analysis by propidium iodide staining and flow cytometry for HCT116 cells treated with the indicated drug combinations (ganetespib at 0.04 μM; palbocilib at 10 μM) for 48 h. (**F**) Scratch assay in HT29 cells under CoCl_2_ treatment (50μΜ) to mimic hypoxia for 72 h. (P: palbociclib; G: ganetespib.) Statistical analysis was performed using one-way ANOVA supplemented with Dunnett test. Mean ± SD was shown. *p < 0.05, **p < 0.01, ***p < 0.001, ****p < 0.0001.

Here we tested the combination of CDK4/6 inhibitors and HSP90 inhibitors. Two different HSP90 inhibitors, ganetespib and onalespib, were tested first in the study. As expected, either ganetespib or onalespib alone reduced the expression level of HIF1α (Fig. 5A, B). The addition of CDK4/6 inhibitor, palbociclib, was able to further enhance the HIF1α decrease induced by HSP90 inhibition (Fig. 5A, B). Knockdown of CDK4 with siRNA exhibited a similar effect (Supplementary Fig. S2A). Combination treatment with palbociclib and either of the HSP90 inhibitors showed synergistic inhibition on cell viability in HCT116 cells in both normoxia and hypoxia (Fig. 5C, D). Such synergy was also observed in other colorectal cancer cells (e.g. SW480, Supplementary Fig. S2B, C). Dual inhibition of CDK4/6 and HSP90 significantly increased the sub-G1 population in HCT116 cells regardless of oxygen concentration (Fig. 5E). Combination treatment with palbociclib and ganetespib significantly inhibited HT29 cell migration in CoCl_2_-treated cells where hypoxia is mimicked (Fig. 5F). These results indicate that targeting CDK4/6 in combination with HSP90 inhibition has a similar anti-cancer effect as dual inhibition of CDK1 and HSP90.

CDK4/6 inhibitors have been intensively studied in combination therapies. After palbociclib, two CDK4/6 inhibitors, ribociclib and abemaciclib, were approved as anti-cancer drugs. Meanwhile there have been many efforts in developing HSP90 inhibitors intended for cancer treatment with tolerable toxicity. To further test the translational potential of the dual inhibition strategy, we included the CDK4/6 inhibitor abemaciclib and two other HSP90 inhibitors that were being examined in clinical trials, XL-888 and TAS-116, in this study. In consistence with the results above, XL-888, in combination with palbociclib, exhibited similar inhibition effects on HIF1α expression as well as cell viability (Supplementary Fig. S3). The combination of TAS-116 and palbociclib or abemaciclib markedly reduced the level of HIF1α (Supplementary Fig. S4A, D) and synergistically suppressed cell viability in SW480 colon cancer cells both in normoxia (Supplementary Fig. S4B, E) and hypoxia (Supplementary Fig. S4C, F).

These results not only established the preclinical foundation for potentially testing these drugs in clinical trials, but further confirmed a class effect of CDK4/6/HSP90 dual inhibition in colorectal cancer treatment.

### Anti-tumor efficacy in vivo by combination treatment with palbociclib and ganetespib

**Figure 6:**
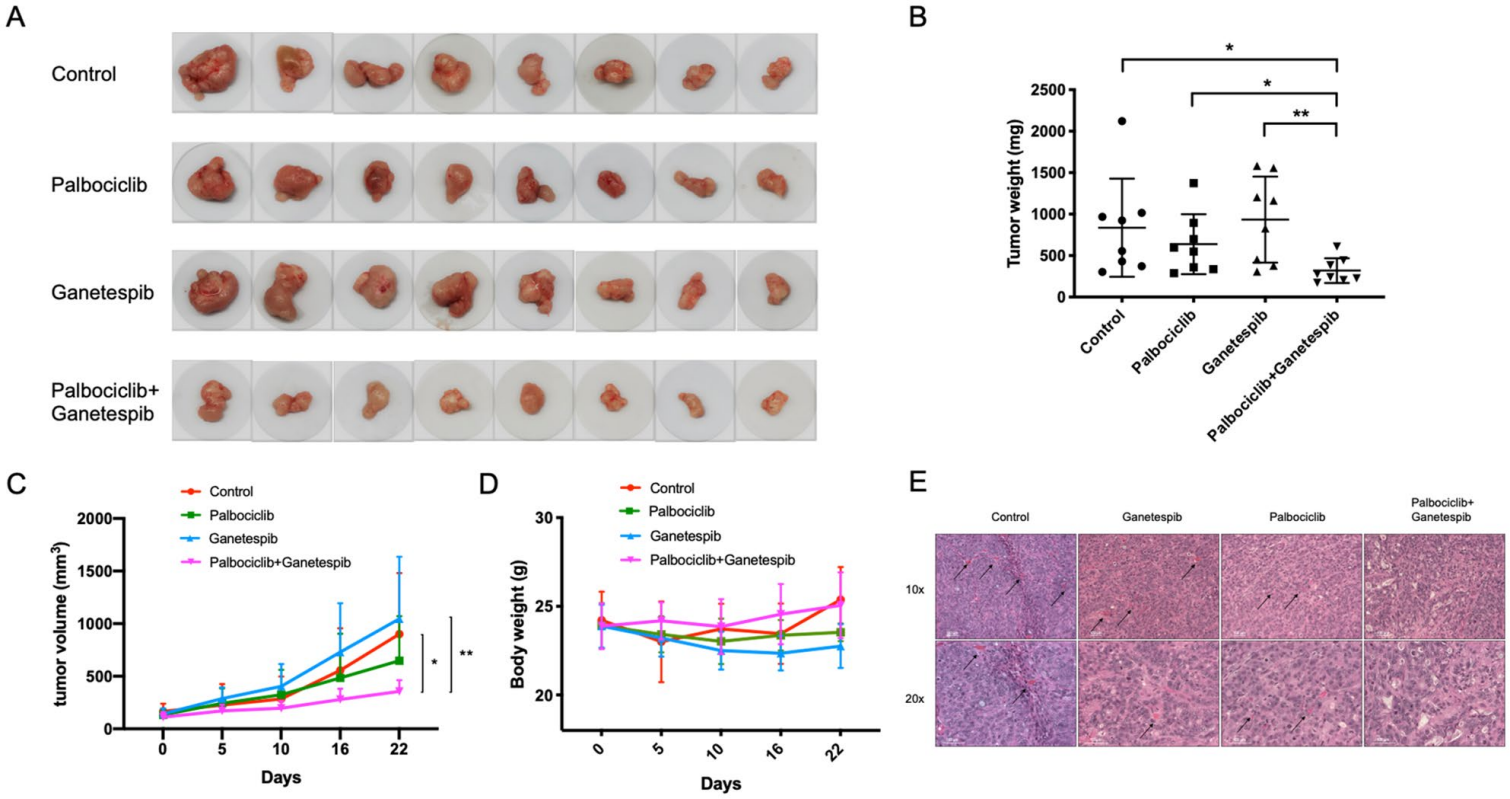
Combination treatment with palbociclib and ganetespib inhibits tumor growth in vivo . (**A**) Tumors excised from HT29 xenografts in nude mice. (**B**) Tumor weight quantification of excised tumors. (**C**) Tumor volume measured over time. (**D**) Body weight of mice in different treatment groups. (**E**) Combination treatment inhibits microvessel formation in tumors in vivo. Statistical analysis was performed using unpaired two-tailed t test. Mean ± SD was shown. *p < 0.05, **p < 0.01.

To determine the anti-tumor efficacy of CDK4/6/HSP90 dual inhibition in vivo, we used HT29 cancer cells in a xenograft mouse model. HT29 is relatively resistant to ganetespib compared to other colorectal cancer cell lines (Supplementary Fig. S5). We tested whether the addition of palbociclib could improve the tumor-suppressive performance of ganetespib. The weight of drug combination-treated tumors was significantly lower than that of control and single treatment groups (Fig. 6A, B). Relative tumor volume was also low in the combination treatment group (Fig. 6C). There was no evident toxicity or weight loss observed upon the combination treatment compared to the control group (Fig. 6D), indicating the safety of simultaneous administration with palbociclib and ganetespib.

We have also analyzed the xenograft study data using a different statistical analysis approach. In the tumor weight analysis, we added tumor weights in the same mouse and took natural log. One-way ANOVA supplemented with Dunnett’s multiple comparisons test showed significant difference between the untreated and combination treatment groups (*p* = 0.0185) as well as between the ganetespib-treated and combination treatment groups (0.0051). In the tumor volume analysis, we modeled the total tumor size (sum of volume of the two tumors on a mouse) for each mouse over time using exponential growth models. Next, we compared the resulting growth parameter estimates across the 4 groups using ANOVA followed by Dunnett's multiple comparison procedures. There were significant differences between the untreated and combination treatment groups (*p* = 0.0481) as well as between the ganetespib-treated and combination treatment groups (0.0108).

Interestingly, the combination treatment reduced the presence of microvessels in tumors (Fig. 6E, Supplementary Fig. S6), which is consistent with suppression of the role of HIF1α in angiogenesis. In addition, the combination treatment increased caspase 3 cleavage and inhibited VEGF expression in the xenografts (Supplementary Fig. S7). The in vivo results suggest a therapeutic potential of the CDK4/6/HSP90 dual inhibition strategy in cancer treatment.

### Combination treatment of CDK4/6 and HSP90 inhibitors synergistically inhibit cell viability in multiple cancer types

Although the dual inhibition was mainly evaluated in colorectal cancers in this study, the strategy is not necessarily limited to one cancer type. CDK4/6 inhibition was initially investigated and approved for treatment in breast cancers. Hypoxia is a prominent characteristic of the tumor microenvironment in pancreatic cancer and glioblastoma, both of which lack efficacious treatments. Thus, we tested the effect of CDK4/6/HSP90 dual targeting on HIF1α expression in various cancer cell lines. In our later studies, we have focused on using TAS-116 as the HSP90 inhibitor as it is currently being tested in early phase clinical trials for cancer. Enhanced HIF1α inhibition was shown upon the combination treatment of palbociclib and TAS-116 in ASPC1 and HPAFII pancreatic cancer cell lines (Supplementary Fig. S8A, B) as well as SKBR3 and MDA-MB-361 breast cancer cells (Supplementary Fig. S8C, D). Palbociclib and TAS-116 synergistically inhibited SKBR3 cell viability in both normoxia and hypoxia (Supplementary Fig. S8E, F). Moreover, ganetespib and palbociclib diminished HIF1α expression in T98G glioblastoma cells (Supplementary Fig. S9A). We have also found that knockdown of CDK4 in combination with HSP90 inhibition inhibited the level of HIF1α in PC3 prostate cancer cell line (Supplementary Fig. S9B). These findings suggest that it may be worthwhile to pursue the translational potential of such combination treatment in more cancer types.

### *Rb*-deficiency does not block the combinatorial inhibition of HIF1α and reduced cancer cell viability is due to targeting of CDK4/6 and HSP90

**Figure 7:**
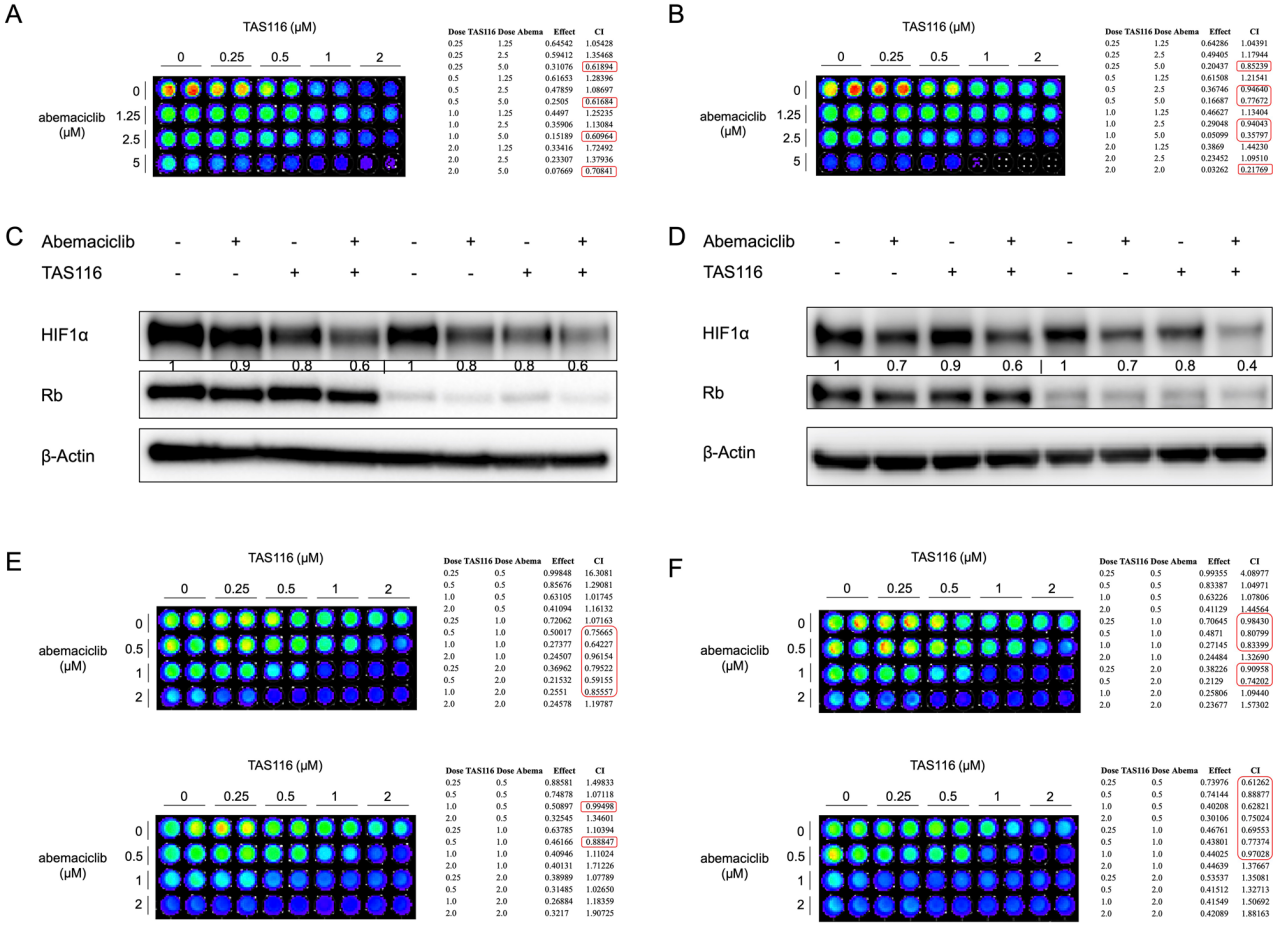
*Rb*-deficiency does not affect the combinatorial inhibition of HIF1α expression and cell viability. (**A**, **B**) Combination treatment with abemaciclib and TAS116 synergistically inhibits cell viability in Saos2 osteosarcoma cells at 72 h under (**A**) normoxia and (**B**) hypoxia (0.5% O_2_). (**C**) SW480 cells were incubated with *Rb*-targeting siRNA for 48 h and subsequently treated with 1 μM abemaciclib and/or 0.5 μM TAS116 for 6 h in hypoxia (0.5% O_2_). (**D**) Knockdown of Rb does not affect HIF1α inhibition by combination drug treatment with TAS116 and abemaciclib in MCF7 breast cancer cells. (**E**, **F**) SW480 cells were treated with (**E**) mock or (**F**) Rb-targeting siRNA for 48 h and subsequently treated with indicated combinations under normoxia (upper) or 0.5% O_2_ hypoxia (lower).

Rb is a key downstream factor of CDK4/6 activity in cell cycle regulation. Loss of Rb protein is believed to convey resistance to CDK4/6 inhibitors. Here we tested whether the inhibitory effect by the combination treatment was diminished by *Rb*-deficiency. Saos2 is an osteosarcoma cell line which is naturally *Rb*-deficient^51^. The combination treatment with abemaciclib and TAS116 synergistically inhibited cell viability at different doses in Saos2 cells in normoxia and hypoxia (Fig. 7A, B). We also knocked down Rb in Rb-proficient (wild-type) cell lines. Knockdown of *Rb* in SW480 cells and MCF7 cells did not affect the inhibitory effect on HIF1α expression upon combination treatment (Fig. 7C, D). The combination treatment also showed synergistic inhibition of cell viability in *Rb*-knockdown SW480 cells (Fig. 7E, F).

### HIF1α transcriptional targets *VEGFA* and *SLC2A1* correlate with poor disease-free prognosis in colorectal cancer

**Figure 8:**
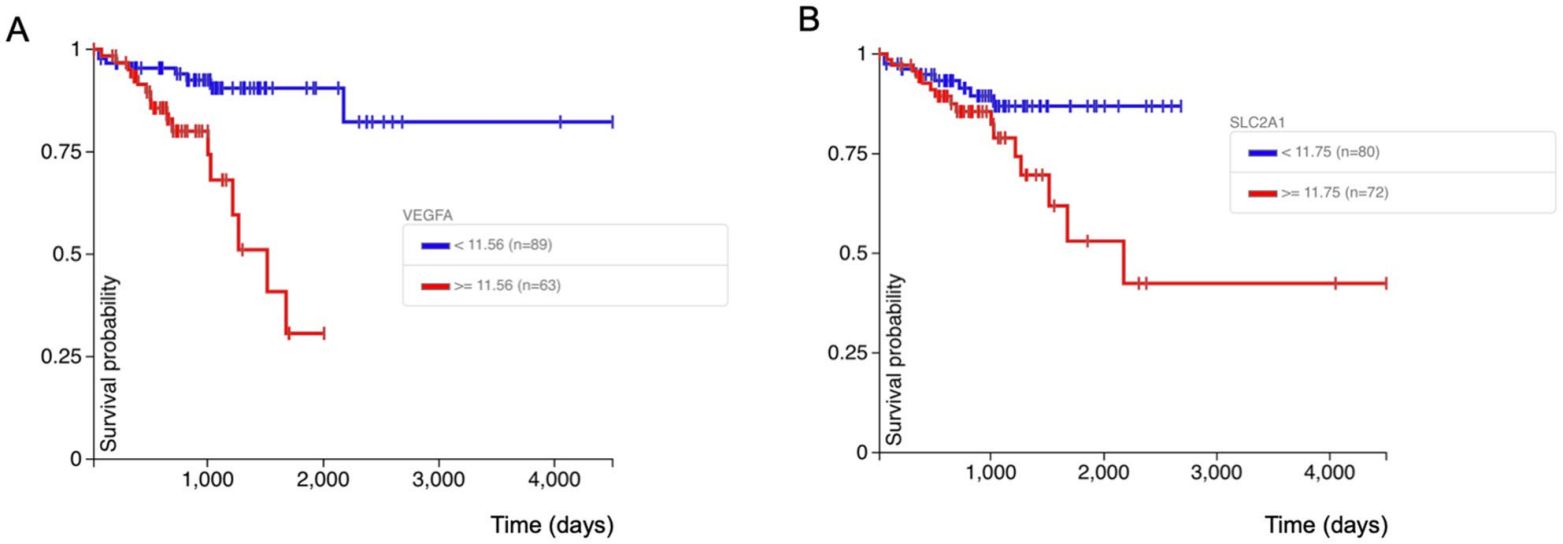
Correlation between the overexpression of HIF1α target genes *VEGFA* and *SLC2A1* and poor disease-free interval. The analysis was performed with the UCSC Xena tool (https://xena.ucsc.edu/) on TCGA colon and rectal cancer samples. (**A**) Correlation between *VEGFA* expression and disease-free interval. *p* value = 0.0001. (**B**) Correlation between *SLC2A1* expression and disease-free interval. *p* value = 0.034.

HIF1α is involved in multiple key signaling pathways in cancer progression. We analyzed the TCGA database on colon and rectal cancer using UCSC Xena online exploration tool. Based on the 2020 TCGA data, the overexpression of HIF1α target genes *VEGFA* and *SLC2A1* correlated with poor disease-free prognosis in colorectal cancer (Fig. 8). Thus, targeting HIF1α may serve as a promising modality in cancer treatment. Here we propose the CDK4/6/HSP90 dual inhibition strategy to suppress HIF1α, which may potentially inhibit those prognostic factors. Both HIF1α and its targets could serve as useful biomarkers in future clinical trials of dual CDK4/6/HSP90 inhibitor therapy.

## Discussion

We demonstrate a novel convergence of CDK4/6 and HSP90 dual inhibition on HIF1α inhibition that is *VHL*-, *p53*-, or hypoxia-independent and which can be translated as a cancer therapy, including for tumors with *Rb*-deficiency. In this regard, the data in this manuscript provides the preclinical rationale for a planned clinical trial combining CDK4/6 inhibitor palbociclib with HSP90 inhibitor TAS-116. The trial is planned for patients with breast cancer who have progressed on CDK4/6 inhibitor therapy and for patients with other solid tumors that are *Rb*-deficient. We are also investigating the link between CDK4/6 and stabilization of HIF1α protein to provide more mechanistic understanding of such targeting strategy. A U.S. patent was issued in 2020 on the use of dual inhibition by CDK and HSP90 inhibitors^52^.

Hypoxia and HIF1α contribute to the malignant cancer progression phenotype across diverse cancer types. HIF1α is hyperactivated and participates in promoting breast cancer progression^53,54^. Anabolic metabolism induced by HIF1α leads to gemcitabine resistance in pancreatic cancer^55^. Also, hypoxia/HIF1α exerts a tumor-promoting role by immunosuppression. Single cell analysis has linked HIF1α inhibition to increased natural killer (NK) cell activity^56^. Depletion of HIF1α in NK cells disturbs angiogenesis and inhibits tumor growth in the MC38 (colon cancer) isograft mouse model^57^. The immune checkpoint protein PD-L1 has been identified as a direct target of HIF-1α^58^. Meanwhile the pro-cancer effect by hypoxia is not limited to solid tumors. Indeed, the local oxygen tension appears quite low in bone marrow in vivo^59^. Hypoxia/HIF1α signaling maintains leukemia stem cells^60^ and facilitates invasion and chemo-resistance^61^ in T-ALL. It may be useful in cancer therapy to pursue effective strategies of targeting hypoxia and HIF1α signaling.

On the basis of our previous findings showing CDK1-mediated stabilization of HIF1α and also with the established role of HSP90 in HIF1α expression, we hypothesized a model where CDK1 contribute to HSP90-mediated stabilization of HIF1α. In our present studies, dual targeting of CDK1 or CDK4/6 and HSP90 robustly reduced the level of HIF1α and synergistically inhibited cell viability in colorectal cancer lines. To assess the anti-tumor effect, the combination of palbociclib and ganetespib was tested on HT29 xenografts. Palbociclib has been used in a colon carcinoma xenograft model at the dose up to 150 mg/kg p.o. once per day to achieve tumor burden suppression^62^. Ganetespib has been used in a HCT116 xenograft colon cancer model at 150 mg/kg i.v. once per week to inhibit tumor growth^36^. In the present study, we administrated into the mice *considerably lower doses* of both compounds (palbociclib at 50 mg/kg; ganetespib at 25 mg/kg). We expect for therapeutic purposes, there would be less toxicity associated with HSP90 inhibition by reduced dosing in this strategy. As the result showed, body weights were not affected by the combination therapy compared to control. However, this does not necessarily preclude the possibility of increasing the doses of each drug in case they are well-tolerated. In the relative tumor volume measurement (Fig. 6C) and tumor weight analysis by one-way ANOVA, although an inhibitory trend was shown by combination treatment, no significant difference was indicated by statistical analysis between the palbociclib alone and the combination groups. This may be due to the accuracy of measurements, variation among individual subjects and limited numbers of animals per group. Notably, HT29 is a relatively resistant cell line to ganetespib. The combination with palbociclib sensitized the xenografts for ganetespib treatment. Combination of palbociclib and ganetespib did not trigger synergistic toxicity to WI38 normal cells in normoxia in vitro (Supplementary Fig. S10). Recently, efforts have been made to develop new generation of HSP90 inhibitors, which may contribute alternative choices other than ganetespib itself. We are planning to use HSP90 inhibitor TAS-116 in combination with palbociclib in the clinical study based on the rationale provided in this manuscript.

In supplementary Fig. S2A, the HSP90 inhibitor, ganetespib, slightly suppresses the CDK4 expression level. It has been shown previously that CDK4/6 inhibitors dissociate CDK4/6 from HSP90-cdc37^63^. Dual targeting of HSP90 and CDK4/6 may simultaneously inhibit the kinase activity while inducing the protein degradation and thus enhance the drug efficacy. There is a possibility that the mechanism of the combination effect by dual inhibition of CDK4/6 and HSP90 also affects HIF1α destabilization by reducing CDK4/6 expression.

Due to the involvement of HIF1α in multiple aspects in cancer biology, whether the combination treatment affects other HIF1α-mediated cancer phenotypes remains to be tested. For instance, HIF1α plays an essential role in stem cell-induced target cell invasion^64^. Hypoxia/HIF1α is shown to regulate cancer stem cell-like features^65,66^. It is not clear whether the CDKi (CDK inhibition) plus HSP90i (HSP90 inhibition) treatment modulates cancer stemness. Also, the effect of combination treatment on metastasis remains to be unraveled, considering the function of HIF1α as a driving force for metastasis/invasiveness^14,67–69^. In addition, since hypoxia/HIF1α is implicated in many immunosuppressive mechanisms^70–73^, it will be of interest to determine whether the combination CDKi/HSP90i treatment modulates the immune response for anti-tumor activities. CDK inhibition has recently been shown to stimulate tumor immune response^74–76^. Furthermore, it remains undefined whether any predictive biomarker(s) could be used to indicate sensitivity to the combination CDKi/HSP90i treatment. In this regard, HIF expression and HIF targets are prime candidate biomarkers. The enhanced inhibition of HIF1α by combined targeting of CDK1 or CDK4/6 and HSP90 was observed in multiple tumor cell lines. It would be useful to explore the anti-cancer effect of combination CDKi/HSP90i treatment in additional cancer types, and based on our results, we plan to include *Rb*-deficient solid tumors in the phase 1b study. The upcoming protocol at Brown University Oncology Group and Lifespan Cancer Institute that has been under development as a result of this research is entitled "BrUOG 387: Phase Ib investigator-initiated trial of heat shock protein 90 inhibitor TAS-116 (pimitespib) combined with cyclin-dependent kinase 4/6 inhibitor palbociclib in advanced breast cancer progressing on palbociclib and treatment-refractory solid tumors with retinoblastoma (*Rb*) deficiency".

We performed a preliminary test on HIF2α expression. The combination treatment slightly reduced the level of HIF2α in HCT116 cells at 6 h (Supplementary Fig. S11). It may be interesting to investigate the effect on HIF2α according to its role in different cancer types (*e.g.* clear-cell renal cell carcinoma).

To test the involvement of HIF1α in the combination treatment, we transiently transfected HCT116 cells with plasmids containing HA alone or HA-*HIF1α*^668E^, a *HIF1α* mutant that remains stable upon CDK inhibition^23^. Overexpression of *HIF1α*^668E^ partially rescued the synergistic effect of cell viability inhibition by combination treatment under hypoxia (Supplementary Fig. S12), indicating that HIF1α may play a role in the combination effect. Since E2F signaling serves as an indicator of CDK4/6 activity, we performed a Pearson correlation test on some of the HIF1α and E2F target genes using the GEPIA tool based on TCGA colon adenocarcinoma data, and found weak correlations between the expression of some HIF1α and E2F targets (Supplementary Fig. S13, S14), which suggests that CDK4/6 activity may be associated with HIF1α signaling in patient tumors. As both Rb and HIF1α are molecular substrates for CDK4/6, we would suggest that HIF1α is a relevant and important target for CDK4/6 inhibitor therapy. In that context, HIF1α and its transcriptional targets may serve as useful biomarkers for drug efficacy.

In summary, we provide a rationale for targeting HIF1α through a novel combination of CDK and HSP90 inhibitors as a potential therapeutic strategy. Our findings suggest new applications of previously approved CDK4/6 inhibitory drugs and novel HSP90 inhibitory agents in combination therapies in multiple cancer types including *Rb*-deficient tumors.

## Materials and methods

### Cell culture

HCT116, SW480, HT29, DLD1 and RKO cells were obtained from American Type Culture Collection. HCT116, HT29 and SKBR3 cells were maintained in McCoy’s 5A medium (Hyclone) with 10% fetal bovine serum (FBS, Hyclone) and 1% penicillin/streptomycin (P/S). SW480, DLD1, RCC4, ASPC1, HPAFII, and T98G cells were maintained in Dulbecco’s modified Eagle medium (Hyclone) with 10% FBS and 1% P/S. RKO cells and PC3 cells were maintained in RPMI 1640 medium (Hyclone) with 10% FBS and 1% P/S. MDA-MB-361 cells were maintained in DMEM-F12 with 10% FBS, 1% P/S and 1% glutamine. Saos2 cells were maintained in McCoy’s 5A medium with 15% FBS and 1% P/S. WI-38 cells were maintained in Eagle's Minimum Essential Medium with 10% FBS and 1% P/S. Cells were regularly tested for mycoplasma and authenticated. All cell lines were maintained at 37 °C in 5% CO_2_. As for hypoxia treatment, cells were kept in a hypoxia chamber (In vivo2, Ruskinn) which maintains 0.5% O_2_.

### Antibodies and reagents

HIF1α (#610958) and Ran (#610341) antibodies were purchased from BD Biosciences. CDK1 (#sc-54), CDK4 (#sc-260) and VEGF (#sc-152) antibodies were purchased from Santa Cruz Biotechnology. HA (#3724S), Rb (#9309S), HSP90 (#4877S), PARP (#9542S), cleaved PARP (#9546S) and cleaved caspase-3 (#9661S) antibodies were purchased from Cell Signaling Technology. Actin (#A5441) antibody was purchased from Sigma. HIF2α (#NB100-122SS) antibody was purchased from Novus Biologicals. MG-132 was purchased from Sigma. Ro-3306 was purchased from Santa Cruz Biotechnology. PD-0332991 (palbociclib) was purchased from Medkoo Biosciences. Geldanamycin was purchased from Invivogen. Ganetespib was purchased from ApexBio or Medkoo Biosciences. Onalespib was purchased from Cayman Chemical Company. XL888 was purchased from Medkoo Biosciences. TAS-116 was purchased from Active Biochem.

### Western blot

Treated cells were lysed in RIPA buffer (Sigma). Protein concentrations were determined using a BCA Protein Assay Kit (Life Technologies). Equal amounts of total protein were boiled with NuPAGE™ LDS sample buffer (Thermo Fisher Scientific) and reducing agent (Invitrogen) or 2-Mercaptoethanol. Samples were analyzed with SDS-PAGE. Proteins were transferred to an Immobilon-P PVDF membrane (EMD Millipore). Primary and secondary antibodies were added in order. Signals were detected after addition of the ECL western blotting substrate (Thermo Fisher Scientific). Images were cropped in Preview or Microsoft PowerPoint. The band intensity was quantified using Image J, compared to internal control and normalized to the average of control lane(s).

We have included original blots for 32 from the total 40 blots in the main figures and original blots for 32 from the total 32 blots in the supplementary figures. For Figs. 1B and 3D we do not include original blots because they are unavailable. The un-cropped images for Fig. 1B (now Fig. 1B Experiment 1) are not available. We always cut the blots to save the amount of antibody needed for membrane incubation. We have found the original blots of a repeated experiment (original blots provided in Supplementary Material) and included the experimental results as Experiment 2 in Fig. 1B. The membranes were cut prior to antibody incubation. In some figures, the shape/edge of the membranes was visible in the original blot images. In others, the membrane background was clean and the probed signal was strong, the shape/edge was not visible within the normal exposure range for respective probed proteins. Regarding those blots, we have provided additional images after each one with approaches such as overexposure, adjusted contrast, or image with visible markers in order to understand where the membrane edge is relative to the protein band signals. As Supplementary Material, we provide images that are clearly labeled and include the corresponding figure panels from the manuscript next to the original images for easy match. We put a border around the relevant parts of blots that were used for figures in the paper and these are shown next to the actual labeled panels for easy comparison. No borders were included in cases where the entire blot was used for a particular panel.

### Cell transfection

Transient transfection of DNA was performed using Opti-MEM (Thermo Fisher Scientific) and Lipofectamine 2000 (Life Technologies). pcDNA3-HA-*HIF1α* plasmid was a gift from William Kaelin (Addgene plasmid #18949)^77^. Knockdown experiments were performed with Opti-MEM and Lipofectamine RNAiMAX (Life Technologies), according to the manufacturer’s protocol. Control, CDK1 and CDK4 siRNAs were purchased from Santa Cruz Biotechnology. Rb siRNA was purchased from Cell Signaling Technology.

### Immunoprecipitation

HCT116 cells were transiently transfected with pcDNA3-HA-*HIF1α*. After 24 h, cells were treated in hypoxia for 6 h with MG132 (1 μM). Cells were washed with PBS and fixed in 4% formaldehyde. Cell lysis was performed in RIPA buffer with gentle sonication. The protein concentration in the lysates was measured and equalized. Part of the lysate was analyzed by SDS-PAGE and western blot for input monitoring. The remaining majority of the lysate was incubated with anti-HA antibody overnight at 4 °C, followed by precipitation with Protein A/G Ultra link Resin (Thermo Fisher Scientific) for 2–4 h.

### Synergy analysis

Indicated cells were seeded in a 96-well black microplate (Greiner Bio-One) and treated with combinations of inhibitors at various concentrations for 48 or 72 h in normoxia or hypoxia. CellTiter-Glo reagent (Promega) was added and mixed on an orbital shaker at room temperature. Luminescence was recorded as a readout to compare viable cell number difference. Combination index between two treatments was calculated using Compusyn software. Synergism was indicated by a combination index value of < 1.

### Colony formation assay

Cells were seeded at the concentration of 500 cells/well in a 6-well plate and allowed to attach overnight. After subsequent drug treatment for 72 h, the culture media was substituted with fresh drug-free complete media. Cells were kept in culture for one to two weeks with medium replacement every three days. At the endpoint, cells were rinsed with PBS and fixed with 10% formalin for 15 min. 0.05% crystal violet was used to stain the colonies. Plates were rinsed carefully in the sink with tap water and let dry at room temperature.

### Sub-G1 analysis

HCT116 cells were treated with indicated reagents for 48 or 72 h in normoxia or hypoxia. Culture media including floating cells were collected and combined with trypsinized (Gemini Bio-Products) attached cells. All harvested cells were washed in PBS with 1% FBS. Cells were fixed with cold 70% ethanol at 4 °C. Subsequently, cells were washed, incubated in phosphate citrate buffer, and stained with propidium iodide (Sigma). The percentage of cells with sub-G1 DNA content was analyzed by propidium iodide staining and flow cytometry.

### Wound healing assay

The indicated cell lines were plated in 12-well plates at 80–90% confluence. Scratch lines were made with a 200-μL pipette tip. After washing with PBS, cells were cultured in media containing reagents as indicated. Images were captured at both the beginning and end of the experiment. Gap width was measured in each image. Each treatment group contained three replicates.

### In vivo studies

Animal experiments were approved by the Institutional Animal Care and Use Committee at Fox Chase Cancer Center and followed the Guide for the Care and Use of Laboratory Animals. All methods were carried out in accordance with the Institutional Animal Care and Use Committee at Fox Chase Cancer Center and followed the Guide for the Care and Use of Laboratory Animals. The study was carried out in compliance with the ARRIVE guidelines. Hairless combined immunodeficient (SCID) mice were monitored in the Laboratory Animal Facility at Fox Chase Cancer Center. HT29 cells were subcutaneously injected into both rear flanks of 4-week old mice at 1 × 10^6^/100 μL in Matrigel/PBS. Treatments were started when tumors reached 100–125 mm^3^ as measured by vernier caliper. Tumor-bearing mice were divided randomly into four groups, and treated with vehicle, palbociclib, ganetespib or the combination of both. Palbociclib was administered orally via gavage at 50 mg/kg daily (dissolved in ddH_2_O). Ganetespib was administered intravenously via retro-orbital injection at 25 mg/kg weekly (dissolved in 10% DMSO, 18% Cremophor RH 40, 3.8% dextrose). Growth of tumors was monitored for three weeks. The tumor sizes were measured by a second investigator. The measurement was blinded. Tumor volume was calculated as Volume = 1/2 × Length × Width^2^. At the endpoint, mice were euthanized with CO_2_ inhalation, and tumors were dissected. The fixation, embedding (with Paraffin), sectioning and hematoxylin and eosin (H&E) staining of tumor samples were performed by the Histopathology Facility at Fox Chase Cancer Center.

### Statistical analysis

Results are presented as the mean ± standard deviation (SD). Difference comparisons were performed with Prism software using the Student’s unpaired two-tailed *t* test or one-way ANOVA supplemented with a post hoc multiple test as indicated in figure legends. Statistically significant difference was determined by *p* value < 0.05.

In the tumor weight analysis, we added tumor weights in the same mouse and took natural log. One-way ANOVA approach was used, followed by Dunnett’s multiple comparisons. In the tumor volume analysis, the sum of tumor volume (V) for each mouse measured over time (t) was assumed to follow the exponential model, V(t) = V0 * exp(t * β), where V0 is the volume at time of treatment initiation and β is the growth slope parameter. V0 and β were estimated separately for each mouse by least squares. ANOVA was used to statistically compare mean growth slopes (β’s) across all 4 treatment conditions. Dunnett’s method was used to perform pairwise comparisons with the combination therapy. All test were two-sided with a 5% type I error. Analyses were conducted using R.

### Correlation analysis

Gene expression correlation analysis was performed using the GEPIA web server (http://gepia.cancer-pku.cn/) on colon adenocarcinoma TCGA data. Pearson correlation coefficient was calculated. The Kaplan–Meier plot was generated using UCSC Xena based on TCGA colon and rectal cancer (https://xena.ucsc.edu/).

## Supplementary Information

Supplementary Figures.
