# Supplementary material for: Anti-cancer efficacy including Rb-deficient tumors and VHL-independent HIF1α proteasomal destabilization by dual targeting of CDK1 or CDK4/6 and HSP90
Source: Sci Rep. 2021 Oct 22;11:20871. doi: 10.1038/s41598-021-00150-8 (PMC8536770; doi:10.1038/s41598-021-00150-8)
Supplement: Supplementary file 1 — Supplementary Figures. [file 41598_2021_150_MOESM1_ESM.docx]

**Supplementary info**

**Anti-cancer efficacy including *Rb*-deficient tumors and *VHL*-independent HIF1α proteasomal destabilization by dual targeting of CDK1 or CDK4/6 and HSP90**

Shuai Zhao^1-4^, Lanlan Zhou^1,3-5^, David T. Dicker^1,3,4^, Avital Lev^6^, Shengliang Zhang^1,3-5^, Eric Ross^6^, and Wafik S. El-Deiry^1-7,^*

^1^Laboratory of Translational Oncology and Experimental Cancer Therapeutics, Warren Alpert Medical School, Brown University, Providence, RI, USA.

^2^Pathobiology Graduate Program, Brown University, Providence, RI, USA.

^3^Department of Pathology and Laboratory Medicine, Brown University, Providence, RI, USA.

^4^Joint Program in Cancer Biology, Brown University and Lifespan Cancer Institute, Providence, RI, USA.

^5^Cancer Center at Brown University, Warren Alpert Medical School, Brown University, Providence, RI, USA.

^6^Fox Chase Cancer Center, Philadelphia, PA, USA.

^7^Hematology/Oncology Division, Lifespan Cancer Institute, Providence, RI, USA

*Corresponding author: [wafik@brown.edu](mailto:wafik@brown.edu)

**Supplementary Figure Legends**

**Supplementary figure 1.** Combination CDK1 and HSP90 inhibitor treatment inhibits colony formation in HCT116 cells. (A) In normoxia or (B) In hypoxia (0.5% O_2_), HCT116 cells were treated with the indicated combination treatments under normoxia or hypoxia for 72 hours. Drug-containing media was replaced with regular culture media, and cells were allowed to grow and form colonies in normoxia for 5 days. Statistical analysis was performed using one-way ANOVA supplemented with Dunnett test. Mean ± SD was shown. * p < 0.05. ** p < 0.01. *** p < 0.001. **** p < 0.0001.

**Supplementary figure 2.** Dual inhibition of CDK4 and HSP90 decreases HIF1α level and synergistically inhibits cell viability in SW480 cells. (A) SW480 cells were treated with DMSO or ganetespib (1 μM) after 48 hours of knockdown of CDK4. (B, C) SW480 cells were treated with palbociclib and onalespib at the indicated doses for 72 hours in (B) normoxia and (C) hypoxia (0.5% O_2_).

**Supplementary figure 3.** Combination treatment with the HSP90 inhibitor XL-888 and CDK4/6 inhibitor palbociclib inhibits HIF1α and cell viability in colorectal cancer. (A) HCT116 and (B) HT29 colon cancer cells were treated with indicated inhibitors (XL-888 at 0.05 μM, onalespib at 0.05 μM, and palbociclib at 10 μM) for 6 hours under hypoxia (0.5% O_2_). (C, D) In HCT116 and (E, F) SW480 colon cancer cells, XL-888 and palbociclib synergistically inhibit cell viability under (C, E) normoxia and (D, F) hypoxia (0.5% O_2_).

**Supplementary figure 4.** The combinatorial effect of CDK4/6 inhibitors with HSP90 inhibitors applies to alternative inhibitors in SW480 cells. (A) SW480 cells were treated with the indicated inhibitors (TAS-116 at 0.5 μM and palbociclib at 10 μM) for 6 hours under hypoxia (0.5% O_2_). (B, C) CDK4 inhibitor palbociclib and HSP90 inhibitor TAS-116 synergistically inhibit the viability of SW480 cells in (B) normoxia and (C) hypoxia (0.5% O_2_). (D) SW480 cells were treated with indicated inhibitors (TAS-116 at 0.5 μM and abemaciclib at 10 μM) for 6 hours under hypoxia (0.5% O_2_). (E, F) CDK4 inhibitor abemaciclib and HSP90 inhibitor TAS-116 synergistically inhibit the viability of SW480 cells in (B) normoxia and (C) hypoxia (0.5% O_2_).

**Supplementary figure 5.** Dose response curve of ganetespib in colorectal cancer cell lines. (A) In normoxia or (B) in hypoxia (0.5% O_2_), cells were treated with increasing doses of ganetespib for 72 hours. Null: HCT116 p53^-/-^ colorectal cancer cells.

**Supplementary figure 6.** Quantification of the microvessel formation in xenograft tumors. (A) The sum of the microvessel formation sites in four different views on the tumor were added. (B) Number of formation sites among treatment groups with three representative tumors in each group.

**Supplementary figure 7.** Combination treatment increased caspase 3 cleavage and inhibited VEGF expression in xenograft tumors. (A) Representative IHC staining of cleaved caspase 3. (B) Representative IHC staining of VEGF. IHC staining was quantified with QuPath software using four different views on the same tumor within three tumors in each treatment group. Statistical analysis was performed using one-way ANOVA supplemented with Dunnett test. Mean ± SD was shown. * p < 0.05. ** p < 0.01. *** p < 0.001. **** p < 0.0001.

**Supplementary figure 8.** Dual inhibition of CDK4 and HSP90 inhibits HIF1α and cell viability in multiple cancer types. (A) ASPC1 and (B) HPAFII pancreatic cancer cells as well as (C) SKBR3 and (D) MDA-MB-361 breast cancer cells were treated with the indicated inhibitors (TAS-116 at 0.5 μM, palbociclib at 10 μM and abemaciclib at 1μM) for 6 hours under hypoxia (0.5% O_2_). (E) In normoxia and (F) in hypoxia (0.5% O_2_), CDK4 inhibitor palbociclib and HSP90 inhibitor TAS-116 inhibit the viability of SKBR3 cells.

**Supplementary figure 9.** Dual inhibition of CDK4 and HSP90 robustly decreases the levels of HIF1α in multiple cancer cell types. (A) T98G cells were treated with palbociclib or ganetespib or the combination of both in hypoxia (0.5% O_2_) for 6 hours. (B) PC3 cells were treated with ganetespib for 6 hours under hypoxia (0.5% O_2_) after 48 hours of knockdown of CDK4.

**Supplementary figure 10.** Combination of CDK1 inhibitor Ro-3306 or CDK4/6 inhibitor palbociclib and HSP90 inhibitor ganetespib does not induce cell death in WI38 normal cells in normoxia.

**Supplementary figure 11.** Dual inhibition of CDK4 and HSP90 slightly inhibits HIF2α in HCT116 cells. Cells were treated with the indicated inhibitors (ganetespib at 0.05 μM, onalespib at 0.05 μM, palbociclib and abemaciclib at 10 μM) for 6 hours under hypoxia (0.5% O_2_).

**Supplementary figure 12.** Overexpression of *HIF1α*^668E^ partially rescued the cell viability inhibition by combination of CDK4/HSP90 inhibitor treatment under hypoxia. Cells were transfected with pcDNA3 plasmid carrying (A) HA tag or (B) HA-*HIF1α*^668E^ for 48 hours, and subsequently treated with the indicated drug combinations for 72 hours under hypoxia (0.5% O_2_). (C) HIF1α overexpression by HA-*HIF1α*^668E^ at 48 hours post transfection or 48 hours transfection plus 6 hours 0.5% O_2_ hypoxia treatment.

**Supplementary figure 13**. Pearson correlation analysis between HIF1α and E2F target genes in colon adenocarcinoma. The analysis is performed using the GEPIA online tool (http://gepia.cancer-pku.cn/) based on TCGA colon adenocarcinoma data.

**Supplementary figure 14.** Analysis of TCGA data in pancreatic adenocarcinoma (PAAD). (A) Overexpression of HIF1α target genes SLC2A1 and PPIA is correlated with poor overall survival and disease-free survival in PAAD patients. (B) Correlations between HIF1α target genes (*SLC2A1* and *PPIA*) and E2F target genes (*RRM2*, *CCNA2* and *CDC6*) based on TCGA PAAD data.
